# Transfer of rhodamine-123 into the brain and cerebrospinal fluid of fetal, neonatal and adult rats

**DOI:** 10.1186/s12987-021-00241-8

**Published:** 2021-02-08

**Authors:** Liam M. Koehn, Katarzyna M. Dziegielewska, Mark D. Habgood, Yifan Huang, Norman R. Saunders

**Affiliations:** Department of Pharmacology & Therapeutics, University of Melbourne, Parkville, Victoria 3010 Australia

**Keywords:** Development, Permeability, Pregnancy, P-glycoprotein, PGP, ABC transporter, blood-CSF barrier, Blood‐brain barrier, Placenta, rhodamine-123

## Abstract

**Background:**

Adenosine triphosphate binding cassette transporters such as P-glycoprotein (PGP) play an important role in drug pharmacokinetics by actively effluxing their substrates at barrier interfaces, including the blood-brain, blood-cerebrospinal fluid (CSF) and placental barriers. For a molecule to access the brain during fetal stages it must bypass efflux transporters at both the placental barrier and brain barriers themselves. Following birth, placental protection is no longer present and brain barriers remain the major line of defense. Understanding developmental differences that exist in the transfer of PGP substrates into the brain is important for ensuring that medication regimes are safe and appropriate for all patients.

**Methods:**

In the present study PGP substrate rhodamine-123 (R123) was injected intraperitoneally into E19 dams, postnatal (P4, P14) and adult rats. Naturally fluorescent properties of R123 were utilized to measure its concentration in blood-plasma, CSF and brain by spectrofluorimetry (Clariostar). Statistical differences in R123 transfer (concentration ratios between tissue and plasma ratios) were determined using Kruskal-Wallis tests with Dunn’s corrections.

**Results:**

Following maternal injection the transfer of R123 across the E19 placenta from maternal blood to fetal blood was around 20 %. Of the R123 that reached fetal circulation 43 % transferred into brain and 38 % into CSF. The transfer of R123 from blood to brain and CSF was lower in postnatal pups and decreased with age (brain: 43 % at P4, 22 % at P14 and 9 % in adults; CSF: 8 % at P4, 8 % at P14 and 1 % in adults). Transfer from maternal blood across placental and brain barriers into fetal brain was approximately 9 %, similar to the transfer across adult blood-brain barriers (also 9 %). Following birth when placental protection was no longer present, transfer of R123 from blood into the newborn brain was significantly higher than into adult brain (3 fold, p < 0.05).

**Conclusions:**

Administration of a PGP substrate to infant rats resulted in a higher transfer into the brain than equivalent doses at later stages of life or equivalent maternal doses during gestation. Toxicological testing of PGP substrate drugs should consider the possibility of these patient specific differences in safety analysis.

## Background

The important role that adenosine triphosphate binding cassette (ABC) efflux transporters play in the distribution of drugs, toxins and endogenous molecules around the body is well known. PGP has been the most widely studied ABC transporter member, shown to efflux a wide variety of substrates at many different barriers of the body (Reviewed in: [1]). PGP efflux at the blood-brain barriers can greatly restrict molecular entry into the central nervous system (CNS) [2, 3]. Drugs that are not substrates for PGP are more likely to be able to reach sites of action within the CNS, unless excluded by other ABC transporters, whereas drugs that do bind to PGP are less likely to have off-target CNS side effects [4, 5].

Recently, studies have identified many complexities regarding the functional capacity of PGP. Two different barriers in the body (e.g. blood-brain barrier and placental barrier) may efflux PGP substrates at different rates [6, 7]. In addition, there may be variation in the level of substrate efflux that occurs at the same barrier (e.g. blood-brain barrier) between two people [6, 7]. Factors such as genetics, disease and medication use can all alter the degree of PGP efflux that will occur for different patients [1, 8–10]. Age can also contribute to the ABC efflux capacity of certain barrier interfaces. At the blood-brain barrier (BBB) of humans and rodents the level of PGP (*abcb1*) increases over the course of development [11–13]. Our recent studies have shown that the percentage of radiolabelled PGP substrate digoxin (^3^H-digoxin) that transferred from blood to the brain and CSF was greater in fetal and newborn rats than in adults [7]. For fetal pups the placenta restricted the amount of digoxin that entered the fetal blood, meaning that although transfer from blood to brain was high the total transfer from maternal blood to fetal brain was similar to the transfer from adult blood to brain [7]. These results have important implications for the understanding of PGP substrate transfer into the developing brain following administration to pregnant women or newborn children. Digoxin does not solely bind to the PGP transporter but also to other transport systems such as Organic Anion Transporting Polypeptide 2 [14]. Replicating these findings with other PGP substrates would strengthen our understanding of whether multiple PGP binding molecules follow a similar developmental profile, especially as juvenile stages of development have not been investigated with digoxin and therefore it is not known at what stage PGP substrate transfer into the brain and CSF matures to adult levels.

Rhodamine-123 (R123) is a naturally fluorescent 380 Da molecule that has been used as a prototypical substrate in many studies to assess the function of the PGP transporter [15–21]. The overlap in substrate affinity of efflux transporters makes complete specificity to one transport system unlikely. However, studies have shown that other transporters such as BCRP and MRP1 are unlikely to have interactions with R123 that compare to the high degree of efflux by PGP [22]. At the placenta PGP is present on the maternal side of syncytiotrophoblast cells, limiting substrate transfer from mother to fetus [23, 24]. Accordingly, the transfer of R123 across the placenta has been shown to be 11 times lower in the maternal-fetal direction than from fetus-mother [25]. At the BBB PGP is located on the blood-facing side of cerebral endothelial cells, restricting molecular transfer from blood to brain [12, 26]. The transfer of R123 into the brain of PGP knockout adult mice has been shown to be 4 fold greater than controls that have PGP at the BBB [15].

In the present study R123 transfer was measured across the E19 rat placenta from mother to fetus and from blood into brain and cerebrospinal fluid (CSF) at E19, P4, P14 and adult ages. These four ages reflect stages of brain development in humans of mid-gestation, late-gestation, newborn children and adults respectively [27]. The results presented describe differences in the transfer of PGP substrate R123 into the brain and CSF at different developmental stages and indicate the importance of placental protection to the overall entry into the fetal brain, as well as the greater vulnerability of the newborn brain.

## Methods

### Animals

Animal experimentation was approved by the University of Melbourne Animal Ethics Committee (Ethics Permission AEC: 1714344.1) and conducted in compliance with Australian National Health and Medical Research Guidelines. The Sprague Dawley strain of *Rattus Norvegicus* was used for this study, supplied by the University of Melbourne Biological Research Facility. Rats were subjected to a 12-hour light/dark cycle with *ad libitum* access to food and water. Age groups investigated were embryonic day 19 (E19), postnatal day 4 (P4), P14 and adult (6–10 week).

### Permeability experimentation

Rhodamine-123 (R123; SIGMA; 2.5–20 mg/kg) dissolved in sterile saline (0.9 % NaCl) was injected i.p. and allowed to circulate for 30 minutes prior to sampling. For postnatal experiments blood samples were taken from the right cardiac ventricle and cerebrospinal fluid (CSF) from the cisterna magna. Brain samples were cortical segments of the frontal and parietal lobes, dissected as previously described [28]. Blood samples were centrifuged (1200*g*, 5 min) to obtain plasma and CSF was examined microscopically to confirm no traces of red blood cells, as previously described [29]. For pregnancy studies the dam was anaesthetized with urethane (SIGMA; 25 %w/v, 1 ml/100 g, i.p.), placed supine on a heating pad (35 °C) and an endotracheal cannula inserted. Blood was sampled from the maternal circulation periodically via a femoral arterial catheter, maintaining blood volume with equivalent volumes of heparinized (Hospira Inc, 5000 international units/ml) saline. Fetal blood, CSF and brain were sampled identical to postnatal procedures. Fetal sampling was conducted from 30 minutes after R123 injection and concluded when placental circulation became insufficient, as previously described [7]. All samples were stored at − 20 °C until use.

### Spectrofluorimetry

**Fig. 1:**
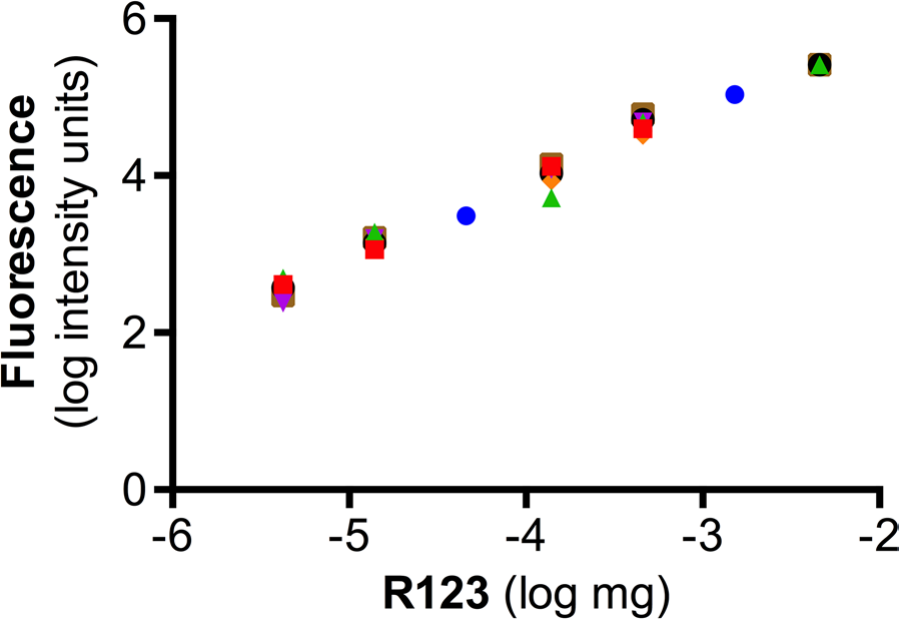
Linear range of R123 detection. Clariostar fluorescence readings of rhodamine-123 (R123) measured as sample extracts in 30 µl Corning microplate (see Methods). Samples analyzed were P4 plasma (blue circle), P14 plasma (red square), adult plasma (green triangle), P4 brain extract (purple inverted triangle), P14 brain extract (orange diamond), adult brain extract (black large circle) and adult CSF (brown square). Note the similarity (overlap) of samples over the linear range. Representative equation for all data points: y = 0.96x + 7.8; R^2^ = 0.98

R123 fluorescence levels were measured using the Clariostar plate reader (BMG Labtech). Samples were loaded onto black Corning 364 well clear bottom plates (04214038). Fluorescence readings were taken at excitation 504 nm and emission 547, as determined by the Claristar spectral analysis setting to identify optimal sample fluorescence. R123 was extracted from cortical samples in 10:1 volume/weight HCl (0.1 M) by manual crushing, pipette dissociation, sonification and vortexing. Extracts were centrifuged (10,000*g*, 5 min) and clear supernatant sampled for analysis. Plasma and CSF samples were measured in 1:5 dilutions. Control samples (from animals not injected with R123) were run on every plate for background correction. Fluorescence readings were converted to R123 concentrations (ng/µl) via standard concentration lines, ensuring measurements were in the linear range (see Fig. 1). Linear range of detection was determined by spiking serial dilutions of R123 into plasma, CSF and brain samples from control animals. R123 was extracted from brains and CSF/plasma samples were diluted for measurement as described above.

### Statistics

Results are described as concentration ratios between compartments of interest (e.g. [brain]/[plasma] ratio) at the time of sampling, as previously described [7, 30]. Shapiro Wilk tests were used to determine data normality, which indicated that non-parametric analysis best suited the data. Statistical differences between concentration ratios were determined using Kruskal-Wallis tests with Dunn’s correction for multiple comparisons. Least squares linear regression with analysis of covariance was conducted as previously described [31]. Significance levels were set at p < 0.05.

## Results

### Linear range of detection

To establish the linear range of R123 detection in blood, CSF and brain samples R123 was spiked into the control tissue/fluid (see Methods). Results are shown in Fig. 1. Least squares linear regression with analysis of covariance indicated that a single line best represented the data. There were no significant differences (p > 0.05) between any two tissues or ages for line elevation or slope. This indicated that R123 fluorescence readings were independent of sample. Therefore, one equation was used to convert fluorescence readings to concentration of R123 (see Fig. 1). The linear range of detection was 4.22 ng-4.59 µg per 30 µl well. This equates to 380 nM to 403 µM of R123 per diluted sample.

### R123 transfer into the postnatal brain

**Fig. 2:**
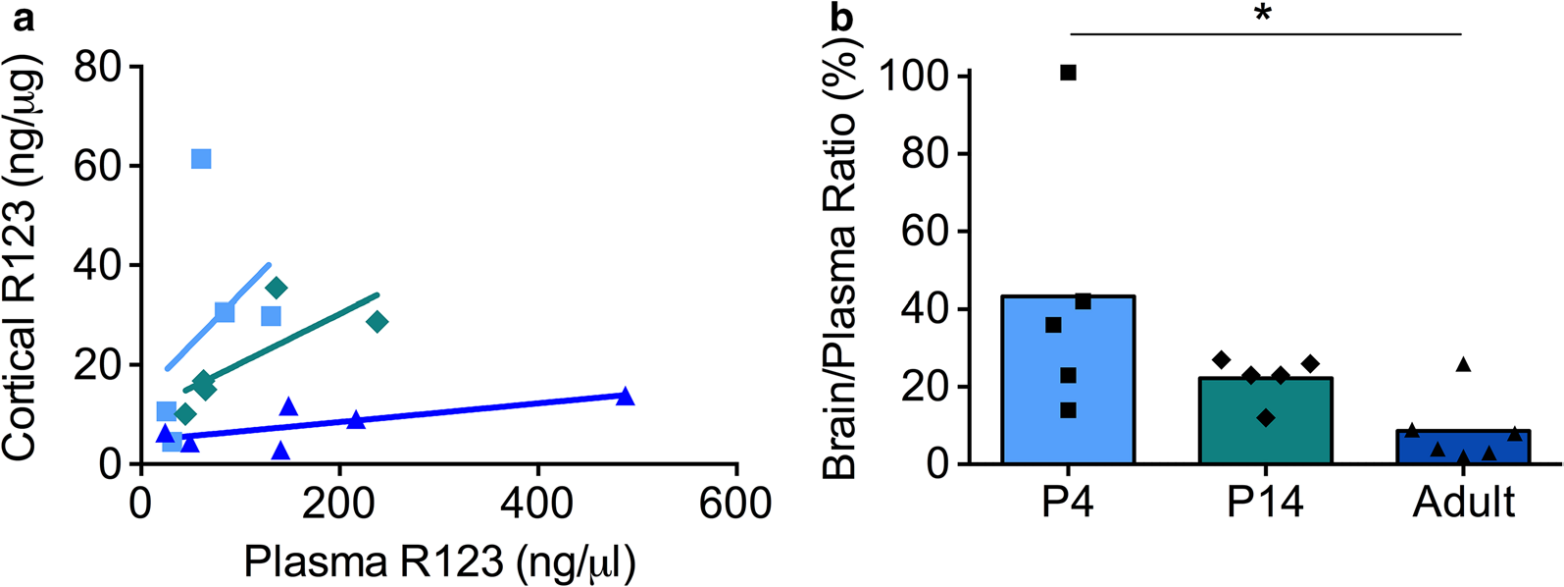
Transfer of R123 into the postnatal brain. **a** The concentration of R123 in the brain cortices compared to the concentration in the plasma (blood). The three age groups investigated were P4 (light blue, squares), P14 (teal, diamonds) and adult (dark blue, triangles) rats. **b** The concentration ratio between R123 in the brain and plasma. Graph **A** and **B** display data from the same rats. Significant differences between age groups are indicated: * p < 0.05 (Kruskal-Wallis with Dunn’s correction)

The concentration of R123 in the plasma and brain of newborn (P4), early neonatal (P14) and adult (6–10 week) rats is shown in Fig. 2. The concentration of R213 in the brain was higher for early postnatal animals (P4, P14) than adults over the range of plasma concentrations investigated (Fig. 2a). Least squares linear regression with analysis of covariance analysis showed that there was a significant difference in the elevation of P14 and adult data (Fig. 2a; p < 0.01). The brain/plasma concentration ratio for all samples was not significantly different between P4 43.3 % ± 34.2 and P14 22.2 ± 5.9 (Fig. 2b). The brain/plasma ratio for adults 8.7 % ± 9.1 was significantly lower than at P4 (0.20 fold, p < 0.05). Adult values were lower than at P14 (0.39 fold) but this did not reach significance (p > 0.05).

### R123 transfer into the postnatal CSF

**Fig. 3:**
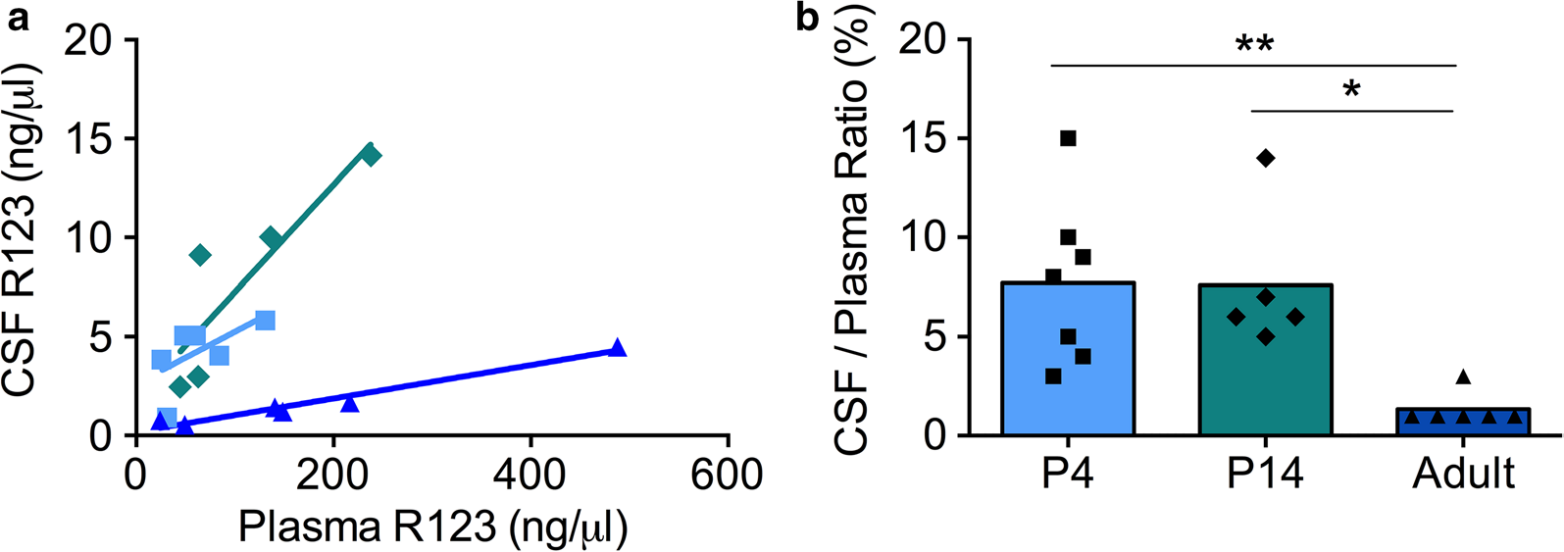
Transfer of R123 into the postnatal cerebrospinal fluid (CSF).** a** The concentration of R123 in the CSF compared to the concentration in the plasma (blood). The three age groups investigated were P4 (light blue, squares), P14 (teal, diamonds) and adult (dark blue, triangles) rats. **b**) The concentration ratio between R123 in the brain and plasma. Graph **a** and **b** display data from the same rats. Significant differences between age groups are indicated: * p < 0.05 and ** p < 0.01 (Kruskal-Wallis with Dunn’s correction)

The concentration of R123 in the plasma and CSF of newborn (P4), early neonatal (P14) and adult (6–10 week) rats is shown in Fig. 3. Similar to the brain, the concentration of R123 in the CSF was higher for early postnatal animals (P4, P14) than adults over the range of plasma concentrations investigated (Fig. 3a). Least squares linear regression with analysis of covariance stated that the lines were significantly different between P14 and adult rats (slope p < 0.01; elevation p < 0.01) as well as between P4 and adult rats (elevation p < 0.001). The steeper gradient of lines for early postnatal rats compared to adults suggests that the discrepancy in CSF transfer between the ages is likely to be highest at higher doses. The CSF/plasma concentration ratios were not significantly different between P4 7.9 % ± 4.2 and P14 7.5 % ± 3.8 (Fig. 3b). The transfer for adults 1.3 % ± 0.9 was significantly lower than P14 (0.17 fold, p < 0.05) and P4 (0.17 fold, p < 0.01). Comparing Figs. 2 and 3 it can be seen that the lines were much more variable for the brain/plasma ratios than CSF/plasma ratios, where the linear trend was much more consistent for each point. The brain/plasma ratio of R123 was higher than the CSF/plasma ratio for each age (see Y-axis; Figs. 2b and 3b; note difference in Y-axis).

### R123 transfer from maternal blood to fetal brain

**Fig. 4:**
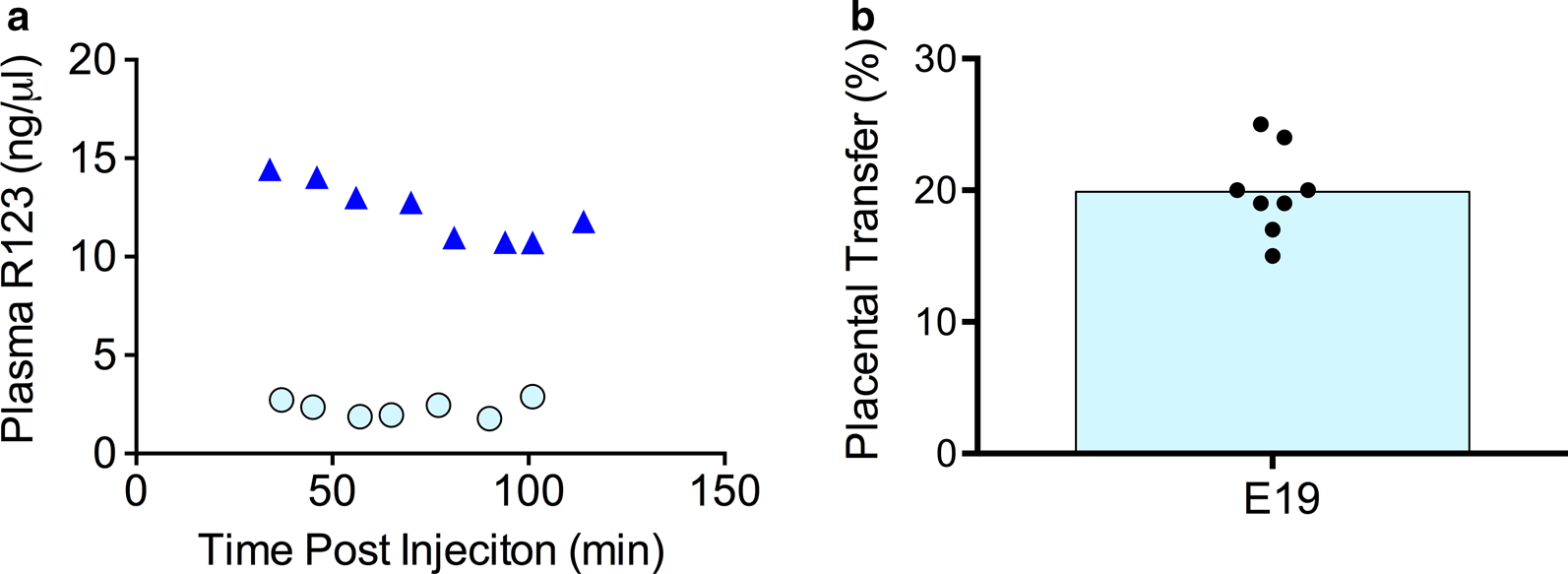
Transfer of R123 across the placenta. Concentration of R123 that reached the fetal blood (plasma) compared to the concentration in maternal plasma, following maternal injection at E19. **a** R123 concentrations in maternal plasma (dark blue triangles) and fetal plasma (light blue circles) over time following maternal R123 injection. Each dot for maternal plasma indicates a serial sample over the course of experiment; each fetal sample indicates an individual pup. **b** Concentration ratios between fetal plasma and maternal plasma for all fetuses over the time course of 30–120 min post maternal injection. Placental transfer (%) is a concentration ratio between R123 in fetal plasma compared to maternal plasma at the time of sampling

Following maternal exposure at E19, the concentration of R123 in the maternal and fetal plasma is shown in Fig. 4. From 30 min to 120 min post-injection maternal plasma levels slightly decreased and fetal plasma levels were reasonably stable (Fig. 4a). Over the course of the experiment R123 transfer across the placenta was restricted, as indicated by the ratio ([fetal plasma]/[maternal plasma]) of 19.9 % ± 3.3 (Fig. 4b).

**Fig. 5:**
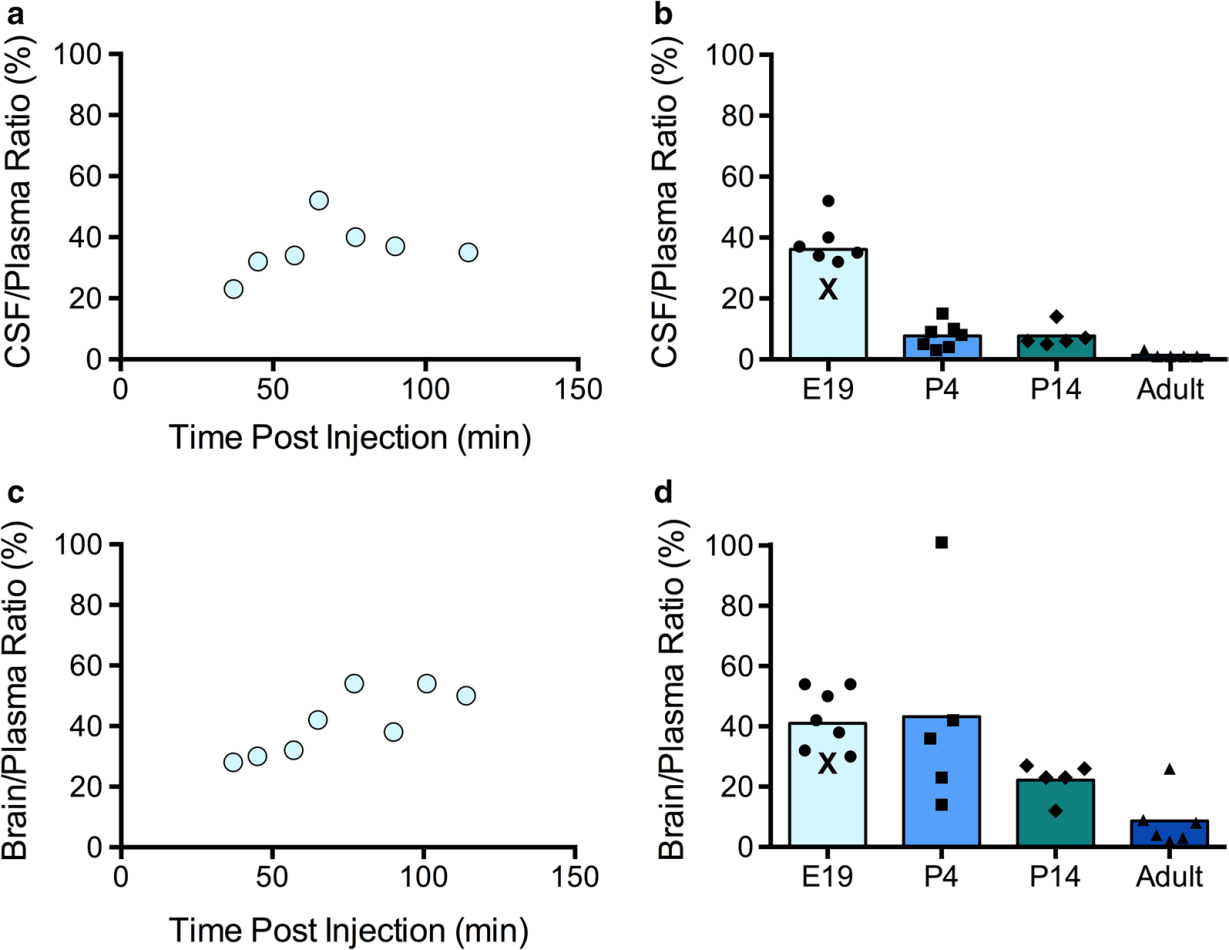
Transfer of R123 into the fetal (E19) brain and cerebrospinal fluid (CSF). Concentration ratios of R123 in the E19 CSF (**a, b**) or brain (**c, d**) compared to E19 plasma (blood) 30–120 min post-maternal injection. The CSF/plasma (**a**) and brain/plasma (**c**) concentration ratios are shown compared to the time post-injection that each individual fetus was sampled (see Methods). For comparison the ratios at E19 are plotted with ratios obtained for P4, P14 and adult rats (**b, d**; data from Figs. 2 and 3). The E19 concentration ratios taken closest to the 30 min post-injection time-point of postnatal values are shown as X. Age is indicated on the X-axis (**b, d**)

Of the concentration of R123 that reached fetal plasma, an average of 38 % ± 7 entered the CSF at the time of sampling (Fig. 5a). This percentage slightly increased from 37 min post-injection (23 %) to 114 min post-injection (35 %). Unlike at postnatal ages, E19 values were not taken at one exact time-point and did not cover the same range of plasma concentrations. However, the CSF/plasma entry for all E19 pups was higher than all postnatal ages, indicating high levels of permeability. On average, the entry of R123 into the E19 CSF was higher than at P4 (5 fold), P14 (5 fold) and adult (26 fold) ages (see Fig. 5b).

The brain/plasma concentration ratio at E19 between 30 and 120 min post dam injection was 43 % ± 10 (Fig. 5c). Similar to transfer into the CSF, brain/plasma ratios increased from 37 min (28 %) to 114 min post injection (50 %). R123 entry into the brain at E19 was similar to that at P4 (0.95 fold) but higher than at P14 (2 fold) and adult (5 fold) ages (see Fig. 5d).

## Discussion

The results from this study show that despite the well-described presence of PGP at the blood-brain barrier [12, 13, 26, 32] the capacity to limit entry of substrates into the brain is developmentally regulated at the ages studied. Transfer of R123 into the brain was highest at early stages of development (fetal, newborn) and decreased through later postnatal stages (P14, adult). Previous studies have suggested that the relative increase of brain and CSF volumes during development [33, 34] can create an apparent decrease in molecular concentrations in the adult CNS due to a larger distribution volume [35]. However, the results described in this study are ratios between concentrations in blood and the brain and because over the course of development the increase in blood volume exceeds the relative increase in brain/CSF volume this cannot fully account for the developmental trends described [33, 36, 37]. The presentation of results over a range of drug concentration in plasma also suggests that the developmental differences are unlikely to be due to variances in the amount of drug that could access the blood from the peritoneum between ages. Previous studies have shown that this developmental trend in BBB transfer is not observed with at least some molecules that pass barriers passively, and are not recognized and effluxed by the PGP transporter [7]. This suggests that the differences in R123 transfer into the brain are likely to be due to differences in PGP capacity at brain barriers rather than being due to developmental factors that influence the transfer of all molecules [7, 28].

**Fig. 6:**
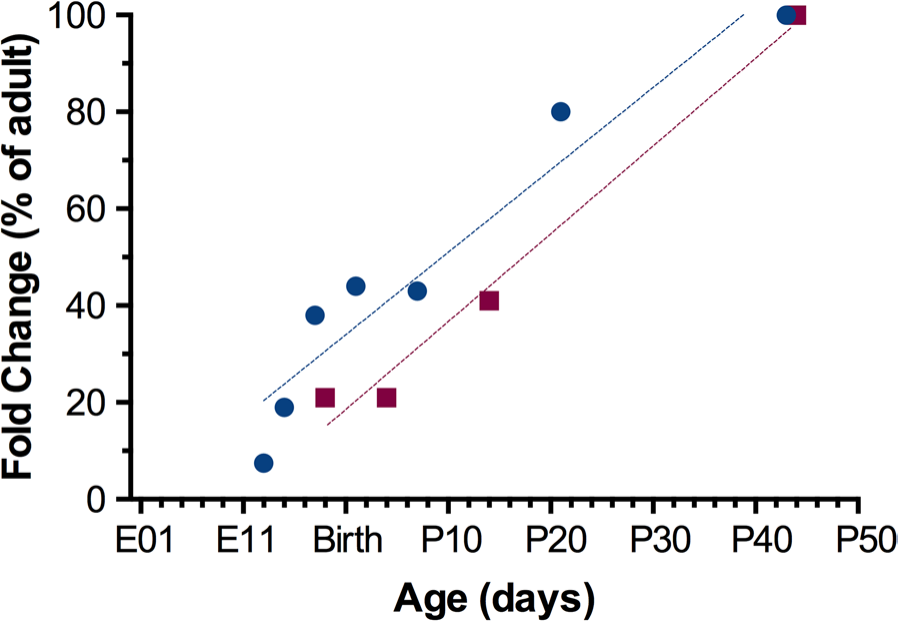
Comparison between R123 transfer into the brain with abcb1a (PGP) levels in the brain at different stages of development. Average concentration ratios for the *restriction* of R123 transfer into the brain ([plasma]/[brain]) at E19, P4, P14 and adult (P42+) are listed as a ratio compared to adult (red squares). Data are the same as Fig. 5. The average level of abcb1a (PGP) in the rat brain at E13, E15, E18, P1, P7, P21 and adult (P42+) as measured in [11], listed as a ratio compared to adult (blue, circles). Age is on the x-axis, with embryonic day (E) before birth and postnatal day (P) after birth. Note the positive gradient both lines, with abcb1a levels in the brain and restriction of PGP substrate R123 entry into the brain both increasing with age. Linear regression lines of best fit are shown for R123 restriction (red; R^2^ = 0.98) and abcb1a levels (blue; R^2   =^0.92)

The age-dependent changes in R123 transfer into the brain correlate with previous reports of PGP levels at the blood-brain barrier. In Fig. 6 the restriction of R123 by the blood-brain barriers measured in this study is compared to the level of PGP gene abcb1a expression in the rat brain, measured in our previous publication [11]. Restriction of R123 transfer into the brain was highest in adults, which corresponds to the highest level of PGP expression [11–13]. PGP levels also increase in the human brain between mid-gestation and adult ages [13], providing some evidence that the functional analysis of ABC transporters at rodent blood-brain barriers may translate to humans. If the functional capacity of PGP is lower at earlier stages of development this should similarly affect the transfer of many other PGP substrates, not just R123. Previous publications have shown a similar percentage transfer profile of PGP substrate digoxin into the brain as was shown by R123 in this study. Transfer of digoxin from blood to brain decreased from 47 % at E19 to 12 % in adults [7], and reflect a similar profile to R123 transfer that was 43 % at E19 and 9 % in adults (Fig. 5). The identification of this age-dependent transfer into the brain using two separate PGP substrates that were measured with different methods (radiolabel, fluorescence) provide strong evidence that PGP functionality at the BBB may change over the course of development. Although these two PGP substrates followed similar percentage profiles, not all PGP substrates may have the same fold changes between ages. Other factors, such as the affinity for the PGP binding site, level of lipid solubility and degree of plasma protein binding, can all contribute alongside changes in PGP functionality to dictate the overall difference in barrier transfer between patients.

Transfer of PGP substrate R123 across the E19 placenta in this study was 20 % (relatively stable over a 30–120 min experimental period). Results from previous studies investigating PGP substrate transfer across the placenta provide varying results depending on the drug selected, route of administration, stage of pregnancy, animal model and time-post injection that measurements were taken. Some studies reported 25–37 % transfer for digoxin and 2.5–25 % for paclitaxel [7, 38, 39]. The overall transfer of R123 from maternal blood into fetal brain in the present study was 9 % (20 % across the placenta, 43 % of that into fetal brain), which was identical to transfer into the adult brain (9 %). This suggests that if pregnant adults take a medication that is a PGP substrate, it is possible that a similar amount of drug could reach both the mother’s and baby’s brains. Previous analysis using similar methodology showed that digoxin followed a similar trend, with 12 % transfer into adult brain and 17 % from maternal blood to fetal brain [7]. R123 transfer differed across multiple barriers in the adult body. Transfer was highest across the placenta (20 %), followed by transfer into the brain (9 %). Transfer into the CSF (1 %) was very low. This may be a product of differences in PGP functionality between the respective barriers or the characteristics of R123 that may predominate in certain tissues/fluids compared to others.

The present study provides a basis on which a host of future analyses could be conducted. The quantification of PGP substrate levels in the brain cortices does not identify whether a substrate was accumulating in the extracellular fluid of the brain, within certain cell types or in subcellular compartments. Future studies may investigate this to better understand what cells within the brain may be at highest risk of PGP substrate-mediated damage at different developmental stages. Metabolomic analysis of PGP substrates may contribute to the understanding of where parent drug molecules and any metabolite forms distribute to following administration. The present study primarily investigated a short time point after i.p. injection (30 min), so a majority of compound in blood would be expected to still be R123 at the time of measurement [40]. In addition, metabolism of R123 has been shown to be negligible during placental transfer [41]. However, deacetylated metabolite rhodamine-110 (R110) may appear in plasma in low amounts [40]. This metabolite is not lipid soluble and is therefore is likely to have low barrier permeability. Investigating changes in the transfer of drug metabolites over the course of development would be an interesting and novel area of research. The present study focused on age as a factor, however similar analysis could be conducted with cohorts of both sexes to identify at what stage(s) of development there may be sex-dependent differences in PGP functionality. A final area of consideration for future studies would be the similar profile of R123 transfer into the CSF and brain over the course of development (Fig. 5), despite a much more prominent change in PGP level at the blood-brain barrier than the blood-CSF barrier [11]. This may be due to age-dependent changes in PGP levels at other interfaces, such as the ventricular CSF-brain [28] that may contribute to a concentration of substrates in the CSF early in development.

## Conclusions

The results from the present study have clinical implications for the use of PGP substrate medications in pregnant women and in newborn children. The placenta provides a degree of protection for substrates entering the fetal bloodstream but higher transfer into the fetal brain may mean that the concentration of drug that reaches both the maternal and fetal brain could be quite similar. The largest differential in drug transfer into the brain compared to adults is likely to be in newborn children. P4 rats have a stage of brain development that resembles a key patient cohort, the earliest viable prematurely born humans, whereas P14 resembles a full-term newborn [27, 42, 43]. Transfer of R123 into the P4 rat brain was 2 times greater than at P14 and 5 times greater than that of adults. Newborn children, particularly those born prematurely, who are without placental protection but still at stages of brain development that have lower efflux transporter capacity than adults may have increased risk of drug transfer to the brain, where damage may occur. Further replication of these results with other PGP substrates, using alternative measurement methods and experimental models, would strengthen the suggestion that the observed developmental trends are indeed due to changes in PGP functionality.

## Data Availability

All data generated or analysed during this study are included in this published article.

## Abbreviations

ABC: Adenosine triphosphate binding cassette
BBB: Blood-brain barrier
CNS: Central nervous system
CSF: Cerebrospinal fluid
E: Embryonic day
i.p.: Intraperitoneal
P: Postnatal day
PGP: P-glycoprotein
R123: Rhodamine-123
